# Data-driven analysis of simultaneous EEG/fMRI using an ICA approach

**DOI:** 10.3389/fnins.2014.00175

**Published:** 2014-07-01

**Authors:** Lena Schmüser, Alexandra Sebastian, Arian Mobascher, Klaus Lieb, Oliver Tüscher, Bernd Feige

**Affiliations:** ^1^Emotion Regulation and Impulse Control Group, Focus Program Translational Neuroscience, Department of Psychiatry and Psychotherapy, Johannes Gutenberg University of MainzMainz, Germany; ^2^Department of Psychiatry and Psychotherapy, Albert Ludwigs University of FreiburgFreiburg, Germany; ^3^Department of Neurology, Albert Ludwigs University Medical CenterFreiburg, Germany

**Keywords:** single-trial EEG/fMRI, trial-to-trial variability, independent component analysis, response inhibition, Go/Nogo, visual response

## Abstract

Due to its millisecond-scale temporal resolution, EEG allows to assess neural correlates with precisely defined temporal relationship relative to a given event. This knowledge is generally lacking in data from functional magnetic resonance imaging (fMRI) which has a temporal resolution on the scale of seconds so that possibilities to combine the two modalities are sought. Previous applications combining event-related potentials (ERPs) with simultaneous fMRI BOLD generally aimed at measuring known ERP components in single trials and correlate the resulting time series with the fMRI BOLD signal. While it is a valuable first step, this procedure cannot guarantee that variability of the chosen ERP component is specific for the targeted neurophysiological process on the group and single subject level. Here we introduce a newly developed data-driven analysis procedure that automatically selects task-specific electrophysiological independent components (ICs). We used single-trial simultaneous EEG/fMRI analysis of a visual Go/Nogo task to assess inhibition-related EEG components, their trial-to-trial amplitude variability, and the relationship between this variability and the fMRI. Single-trial EEG/fMRI analysis within a subgroup of 22 participants revealed positive correlations of fMRI BOLD signal with EEG-derived regressors in fronto-striatal regions which were more pronounced in an early compared to a late phase of task execution. In sum, selecting Nogo-related ICs in an automated, single subject procedure reveals fMRI-BOLD responses correlated to different phases of task execution. Furthermore, to illustrate utility and generalizability of the method beyond detecting the presence or absence of reliable inhibitory components in the EEG, we show that the IC selection can be extended to other events in the same dataset, e.g., the visual responses.

## Introduction

Behavioral variability such as the varying effectiveness of motor inhibition or trial-to-trial variations of reaction times has been linked to single-trial variabilities in neural processes (MacDonald et al., 2006; Fontanini and Katz, 2008; Ledberg et al., 2012). It has been shown that single-trial variability of evoked neural activity can be modeled by a combination of random ongoing network activity and stationary stimulus-related responses (Arieli et al., 1996). Therefore, single-trial fluctuations of evoked responses contain aspects of moment-to-moment fluctuations in the participant’s brain state rather than only representing noise (Lutz et al., 2002; Kelly et al., 2008; Ledberg et al., 2012). Assessing trial-to-trial variations is also key to characterize intra- as well as inter-individual behavioral and neural processing phenotypes.

In order to meaningfully analyze trial-to-trial variations, they have to be related to other parameters available for the same single trials. Trial-by-trial coupling of simultaneous EEG/fMRI data (Debener et al., 2005; Eichele et al., 2005; Huster et al., 2012) allows relating single-trial event-related EEG with single-trial BOLD data in an attempt to map the changes in the participant’s brain state mentioned above to changes in metabolic brain activity. This approach is also designated “integration-by-prediction” regarding that single-trial EEG/fMRI analyses usually employ EEG-derived regressors as predictors of the fMRI BOLD responses (for more detail see Debener et al., 2006; Eichele et al., 2009; Bland et al., 2011). In the process of isolating task-related single-trial EEG activity, different routines have been used: single-trial EEG features are extracted from single independent components (ICs) reflecting best the EEG component of interest (Debener et al., 2005; Feige et al., 2005; Mobascher et al., 2009), from artifact-cleaned EEG data using several electrodes (Eichele et al., 2005; Novitskiy et al., 2011) or single electrodes (Bénar et al., 2007; Mulert et al., 2008; Warbrick et al., 2009; Karch et al., 2010; Scheibe et al., 2010; Juckel et al., 2012; Baumeister et al., 2014). However, in the majority of studies components of interest were identified by visual inspection, which depends on subjective evaluation and can be biased by inter- and intra-individual variations of the evaluator.

Greater objectivity can instead be provided by data-driven approaches of component selection. Goldman et al. (2009) for example selected task-discriminating EEG components in a completely data driven way. The authors identified task-related components which discriminated two task conditions in stimulus-locked and response-locked time windows. They demonstrated that single-trial correlations of these task-discriminating components with fMRI BOLD responses could reveal brain areas different from those yielded by classical fMRI analyses. However, an important limitation of this linear discrimination method is that the algorithm could extract only one EEG component for a given time window and thus could miss meaningful components. Another approach that circumvents this by selecting the components in source space instead of sensor space was presented by Wessel and Ullsperger (2011). The authors developed an algorithm (COMPASS) that automatically identifies ICs contributing to the event-related potential (ERP) of interest by comparing each IC with a predefined ERP template. This approach however is limited by restricting the selection procedure to predefined ERP templates. Thus, we aimed to develop a new approach enabling us to classify and select inhibition-related ICs intra-individually in a data driven way without a priori implicating known ERP components. This approach avoids the analytical bias of the assessment of EEG correlates of task-related activity introduced by the restriction to distinct ERP components. Furthermore, by classifying and selecting ICs intra-individually our approach takes into account known inter-individual differences in neural processing (Kanai and Rees, 2011).

This automated procedure was applied to simultaneously acquired EEG and fMRI data of 39 healthy control participants who had performed a visual Go/Nogo task. This paradigm has been used in several EEG/fMRI studies, including studies investigating the effect of fMRI data acquisition on ERP. Bregadze and Lavric (2006), for instance, showed that Nogo-related ERP components can be extracted from EEG data recorded simultaneously with fMRI data acquisition. In 2011, Lavric et al. demonstrated that the detection of task-related modulations of N2/P3 ERPs could be improved by applying ICA-based analyses. Variants of the classical Go/Nogo task were also used in simultaneous EEG/fMRI studies in order to assess the neural correlates of response inhibition. Using a cued auditory Go/Nogo task during simultaneous EEG/fMRI, Karch et al. (2008) found correlations of fMRI BOLD signal in insular, right temporo-parietal and medial frontal cortex with fronto-central Nogo-P3 amplitude values. Most recently, Baumeister et al. (2014) investigated the role of N2 and P3 in cognitive processes associated with response inhibition by using parametric modulation of fMRI BOLD signal with both N2 and P3 single-trial amplitude values derived from Cz. This analysis revealed an association of N2 with attentional processes while P3 was associated with inhibitory processes but also with memory recollection and internal reflection (Baumeister et al., 2014). Although the above studies hint at a relation of the N2/P3 complex to Go/Nogo inhibitory processes it is not clear how specific variations of the N2/P3 complex are for inhibition. Thus, instead of selecting a-priori defined distinct ERP components such as the N2/P3 complex we used a purely data-driven approach to select ICs reliably associated with inhibition.

**Figure 1:**
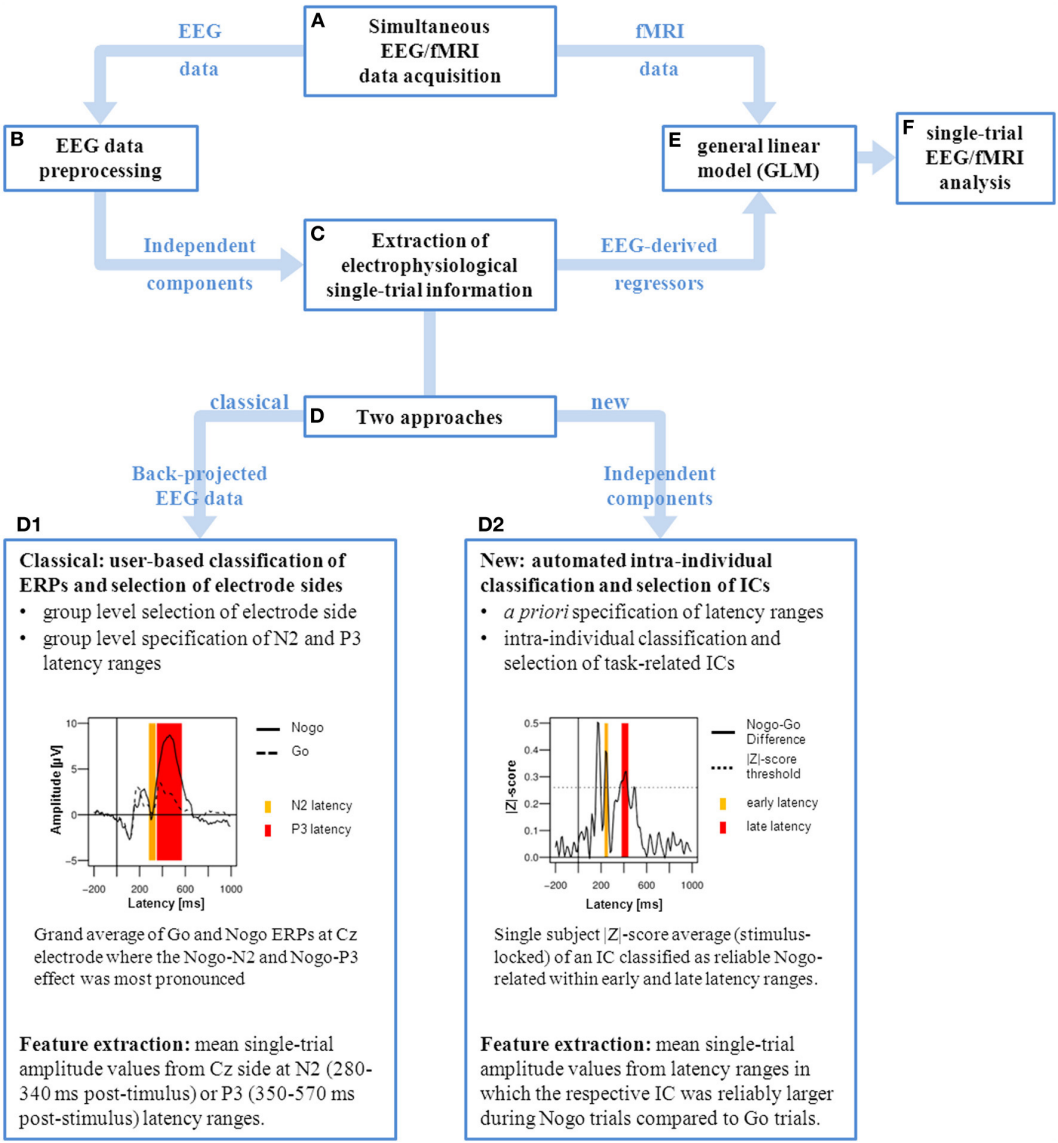
**Graphical representation of single-trial EEG/fMRI analysis**. After simultaneous EEG/fMRI data acquisition **(A)** the EEG data is preprocessed and corrected for fMRI artifacts **(B)** using independent component analysis (ICA). Subsequently the electrophysiological single-trial values can be extracted **(C)** using different approaches **(D)**. Classically **(D1)**, single-trial amplitude values are extracted from predefined ERP components. This is based on a chosen electrode site where the ERP component of interest (Nogo-N2 and Nogo-P3) is most pronounced in the grand mean average. Followed by the specification of N2 (280–340 ms, yellow) and P3 (350–570 ms, red) latency ranges which cover best the task-related ERP effects on group level at the selected electrode site (Cz). For each participant the mean single-trial values are extracted from these predefined latency ranges. Alternatively **(D2)**, our approach allows to extract single-trial values from independent components (ICs) which are intra-individually classified and selected in an automated procedure. This is based on *a priori* specification of latency ranges of interest, in this case located prior (early, yellow) and around (late, red) the individual’s median response time (RT). ICs are intra-individually classified according their association with the Nogo condition (significantly increased amplitudes in Nogo trials compared to Go trials). For each participant the mean single-trial values are extracted from latency ranges in which the respective IC was reliably larger during Nogo. In both approaches the resulting electrophysiological regressors are included in the general linear model of fMRI data analysis **(E)** in order to perform the single-trial EEG/fMRI data analysis **(F)**.

The present study was designed to examine the BOLD correlates of variations in electrophysiological inhibition-related components in a data-driven approach. While previous studies used fixed latency windows and distinct EEG channels to derive regressors from the EEG, relying upon data from other EEG studies or own grand averages for their choice, we automatically selected IC components reliably associated with response inhibition for each single participant. We ensured to use only ICs which had reliably larger amplitude in Nogo than in Go trials securing the specificity of EEG components for neural activity of response inhibition. We therefore introduce a newly developed data-driven analysis procedure that automatically selects participant-specific electrophysiological ICs which are reliably and specifically Nogo-related at an early or late stage of response inhibition to inform fMRI data analysis. To assess and validate the performance and outcome of our automated procedure in the context of combined EEG/fMRI analysis procedures, we compared our automated IC-based approach to an approach based on selecting single-trial amplitude values from predefined ERP components (see Figure 1 for a graphical overview). Thus, in line with Baumeister et al. (2014) we extracted for each participant the mean amplitude values of N2 (280–340 ms post-stimulus) and P3 (350–570 ms post-stimulus) from the Cz site. Second, to illustrate the utility of the method beyond detecting Nogo-related components, we used the same data-driven analysis procedure for detecting visual responses in the same dataset. Visual components are well suited for validation purpose, as consistent results have been found in previous EEG/fMRI studies with different task settings (Di Russo et al., 2002, 2005; Novitskiy et al., 2011; Warbrick et al., 2013). Using separate EEG and fMRI data acquisition, Di Russo et al. (2002, 2005) showed that the P1 and N1 subcomponents can be accounted for by dipoles localized to middle occipital gyrus, fusiform gyrus and parietal lobe. More recently Novitskiy et al. (2011) and Warbrick et al. (2013) found positive single-trial correlations of visual components with fMRI BOLD signal in regions of the visual dorsal stream but also in medial frontal and precentral gyri.

## Materials and methods

Taking advantage of the fact that independent component analysis (ICA) can be used to isolate task-related components (Debener et al., 2005; Bagshaw and Warbrick, 2007), we employed ICA in order to selectively extract time series related to different phases of task execution of a visual Go/Nogo task. Importantly, the algorithm we used was not designed to identify ICs associated with classical event-related components such as N2 and P3, but to automatically select ICs with significantly increased amplitudes in Nogo trials compared to Go trials within predefined time windows located prior and around the individual’s median response time (RT). Thus, for each participant, EEG data were decomposed into temporally ICs which were then intra-individually classified according to their relation to response inhibition (Nogo > Go). ICs identified as reliably Nogo-related within a predefined time window were combined into individual electrophysiological regressors and then included into fMRI first-level analysis.

### Experimental design

#### Participants

Thirty-nine participants (16 males; mean age: 38.85 ± 16.48) were included in this analysis. Participants were recruited from a larger sample (Sebastian et al., 2013a) because of their good overall data quality for EEG and fMRI (see section Data Preprocessing). All participants were right-handed (Oldfield, 1971) and had normal or corrected-to-normal vision. Structural Clinical Interview for DSM-IV Axis I and II Disorders (SCID-I/II) was used (Wittchen et al., 1997) to exclude participants with a lifetime history of axis I or axis II disorders. The study was approved by the Ethics Committee of the University of Freiburg Medical School and all participants gave their informed consent prior to MRI scanning. Each participant received a financial compensation of €55.

#### Experimental paradigm

During the simultaneous EEG/fMRI session all participants performed a visual Go/Nogo-task. Participants were instructed to respond by pressing a mouse button with the right index finger to every letter (Go stimulus) except for the letter “X” (Nogo stimulus). Each letter was shown for 500 ms followed by a black screen for the next 500 ms. Every participant completed two runs each consisting of 300 stimuli. Nogo stimuli were presented with a mean probability of 29%, and each Nogo stimulus was followed by at least one Go stimulus (Sebastian et al., 2013a,b, 2012). Visual stimuli were programmed using the software “Presentation” (Neurobehavioral Systems, Version 11.1 http://www.neurobs.com/) and were projected on a screen at the head end of the scanner bore and viewed with the aid of a mirror mounted on the head coils. Participants had in advance received a brief training session on the task outside the scanner room.

### Data acquisition

The study was conducted at the University Hospital of Freiburg (Department of Radiology). fMRI data acquisition and EEG recordings were initiated manually whereas visual presentation was initiated by a trigger code sent from the MR scanner. The EEG-amplifier hardware clock was synchronized with the timing of gradient switching during fMRI measurements (SyncBox; Brain Products, Gilching, Germany). Onsets of stimulation and echo-planar image (EPI) scans as well as the participant’s response were registered on a trigger channel of the EEG acquisition host.

#### fMRI/MRI

MRI data was collected using a 3T tim-TRIO scanner (Siemens Medical Systems, Erlangen) equipped with a 12 channel head coil. Foam padding was used to limit head motion within the coil. For functional BOLD imaging, T2^*^-weighted EPI volumes were acquired (*TR* = 2250 ms, *TE* = 30 ms, flip angle = 90°, *FOV* = 92 mm, voxel size = 3 × 3 × 3 mm, 36 slices) by applying fully automated PACE (Prospective Acquisition Correction) motion correction (Thesen et al., 2000) and distortion correction based on point spread function mapping (Zaitsev et al., 2004). Per run 157 complete brain volumes were acquired. Following fMRI data acquisition, the EEG cap was removed and 3D MRI data for anatomical references was acquired using a 3D magnetization prepared, rapid acquisition gradient echo (MPRAGE) sequence (*TR* = 2200 ms, *TE* = 4.11 ms, flip angle = 12°, *FOV* = 256 mm, voxel size = 1 × 1 × 1 mm).

#### EEG

Continuous EEG data was recorded with a 64-channel EEG-system consisting of two 32-channel MR compatible EEG-amplifiers (BrainAmp MR plus; Brain Products) powered by a MR-compatible rechargeable battery pack (PowerPack, Brain Products). The EEG system was placed inside the scanner bore directly behind the head coil. This allowed for the use of short wires, thus reducing potential scanner artifacts caused by wires moving inside the magnetic fields. A total of 62 sintered Ag/AgCl ring electrodes were placed within an elastic EEG-recording cap (EasyCap, Falk Minow Services, Herrsching, Germany). Electrodes were placed according to an extended international 10–20 system with reference electrode positioned at FCz and ground electrode positioned at AFFz. In order to monitor electrocardiograms (ECG) and eye blinks (EOG), additional electrodes were placed beneath the participant’s left scapula and below the left eye. Electrode-skin contact impedances were maintained below 10 kΩ. The recorded analog EEG signal was filtered between DC and 1 kHz, digitized with a sampling frequency of 5 kHz and transmitted via fiber optic cables to a recording PC placed outside the scanner room. To facilitate subtraction of the gradient artifact, EEG sampling was driven by the clock board of the MR scanner (SyncBox, Brain Products). The Brain Vision Recorder software (Brain Products) was used to acquire, store and display EEG recordings online.

### Data preprocessing

#### fMRI preprocessing

Image preprocessing was performed using SPM5 (Wellcome Trust Center for Neuroimaging at UCL, London, UK; http://www.fil.ion.ucl.ac.uk/spm/software/spm5) running under Matlab 7.7.0 (The MathWorks Inc., Natick, Massachusetts, USA; http://www.mathworks.com). Images were screened for motion artifacts prior to data analysis. No excessive head motion (>2 mm) was observed in any of the subjects. Next, images were manually reoriented to the T1-template of SPM. To allow for equilibrium effects the first five volumes of each run were discarded. Functional images were then realigned to the first image of the first run (six degrees-of-freedom rigid body transformation) and coregistered to the individual T1. The T1 image was then spatially normalized (linear and non-linear transformation) into the reference system of the Montreal Neurological Institute’s (MNI) reference brain. Functional images were spatially normalized using the resultant normalizing parameters and then smoothed by applying a 3D isotropic Gaussian kernel (8 mm full-width at half maximum, FWHM).

#### EEG preprocessing

EEG data was processed offline using AvgQ (Feige, 1999; Freiburg, Germany; https://github.com/berndf/avg_q), an open source multichannel (EEG/MEG) data processor driven by Python scripts. Gradient artifact correction was performed by template subtraction (Allen et al., 2000). Data upsampling was not necessary since the EEG sampling was synchronized to the gradient clock (SyncBox). In order to remove low-frequency drifts as well as residual scanner artifacts, the gradient-corrected EEG data was then run through a bandpass filter (0.2–48 Hz) and down-sampled to 100 Hz. Afterwards, an unmixing matrix was estimated using the extended infomax algorithm (ICA, Bell and Sejnowski, 1995; Lee et al., 1999; Makeig et al., 1999, 2002) for the continuous EEG data of each participant and run separately. By multiplying the continuous EEG data with this unmixing matrix, IC activations can be computed for any EEG sample without repeating the ICA training. For ballistocardiographic (BCG) and electrooculographic (EOG) artifact detection and correction, averages related to heart beat and eye blink were computed. Single heart beats were detected in the ECG signal by convolution with a time-domain ECG template. The continuous EEG was averaged with respect to the detected heart beats and ICs loading on this average identified. Artifact correction was performed by removing these ICs. Based on the BCG artifact corrected EEG data, EOG artifact detection and correction was performed similarly. Importantly, BCG/EOG artifact corrected EEG is not used in the main method illustrated here. In this method, the electrophysiological information used for single-trial EEG/fMRI analysis is extracted from the IC time courses themselves, excluding those ICs representing BCG/EOG artifacts. Participants of an initially larger sample (Sebastian et al., 2013a) of whom the artifact correction did not achieve usable datasets were excluded from further analysis.

### Classification and selection of independent components

The IC time courses of each participant were segmented into epochs of 1200 ms starting 200 ms prior to stimulus onset. The 200 ms pre-stimulus interval was used for baseline correction. Epochs belonging to the same event type (i.e., correct response: Go and Nogo; incorrect response: omission of Go trials and commission errors in Nogo trials) were averaged, resulting in four different event-related averages each consisting of 64 averaged ICs.

#### Reliability testing and thresholding

In addition to the pointwise mean, pointwise variance information was collected. Together they were used to compute pointwise *t*-tests comparing either the Nogo and Go conditions (two-sample *t*-test for independent groups) or comparing the average against baseline (one-sample *t*-test) on each IC and event type. In a second step, the point-wise *t*-values were transformed into *Z*-scores as basis for the subsequent IC classification. As high *Z*-scores are indicative of high signal-to-noise ratios, only ICs with absolute *Z*-scores crossing a predefined threshold entered further analysis steps. Since there were 300 trials per block of which 90 were Nogo trials, we selected a *Z*-score of 0.275 which corresponds to a two-sided *p*-value of 0.01 (two-sample *t*-test with *df* = 89), choosing the degree of freedom conservatively from the condition with the smaller number of epochs contributing to the analysis.

#### Nogo-related ICs

***IC classification.*** To determine components reliably associated with the Nogo condition, *Z*-score differences between Nogo- and Go-related IC averages were computed as described in section Reliability Testing and Thresholding. Nogo-Go differences were classified as sufficiently reliable if their absolute *Z*-score exceeded a threshold of 0.275. All latency ranges with above-threshold *Z*-scores were noted, i.e., the latencies at which the threshold was crossed in positive and in negative direction. The polarity of ICs in a given latency range is arbitrary. To be able to select only such ICs and latency ranges in which the Nogo amplitude was larger, a direction was defined for each latency range by noting the polarity of the event-related IC average with the larger absolute amplitude in this latency range. For each participant, a list of Nogo-related ICs, latency ranges and polarities was formed keeping only those associated with larger absolute amplitudes in Nogo trials.

**Figure 2:**
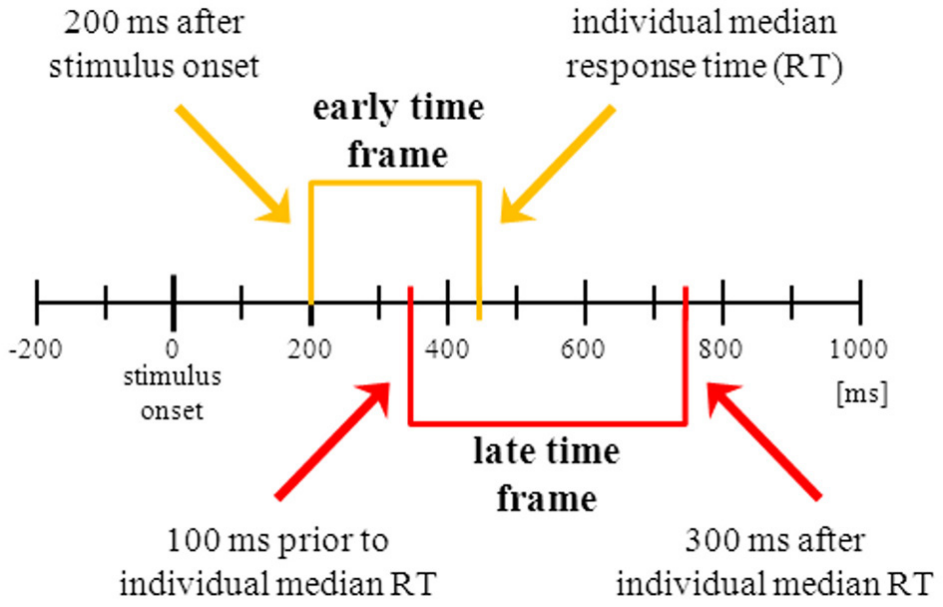
**Visualization of the two predefined time windows in which the independent component’s (IC) latency range of significant activation had to be confined**. Starting point of the “early” time frame (yellow brackets) is 200 ms after stimulus onset, ending point is the individual median correct Go response time (RT). The “late” time frame (red brackets) starts 100 ms prior to RT and ends 300 ms after RT. To be selected, the IC’s latency range of reliable activation must be wholly contained within one of the predefined time windows, i.e., the Nogo minus Go absolute *Z*-score must arise above threshold (|*Z*| > 0.275) and must fall below threshold (|*Z*| < 0.275) within the time window. Black bars depicting 100 ms intervals.

**Figure 3:**
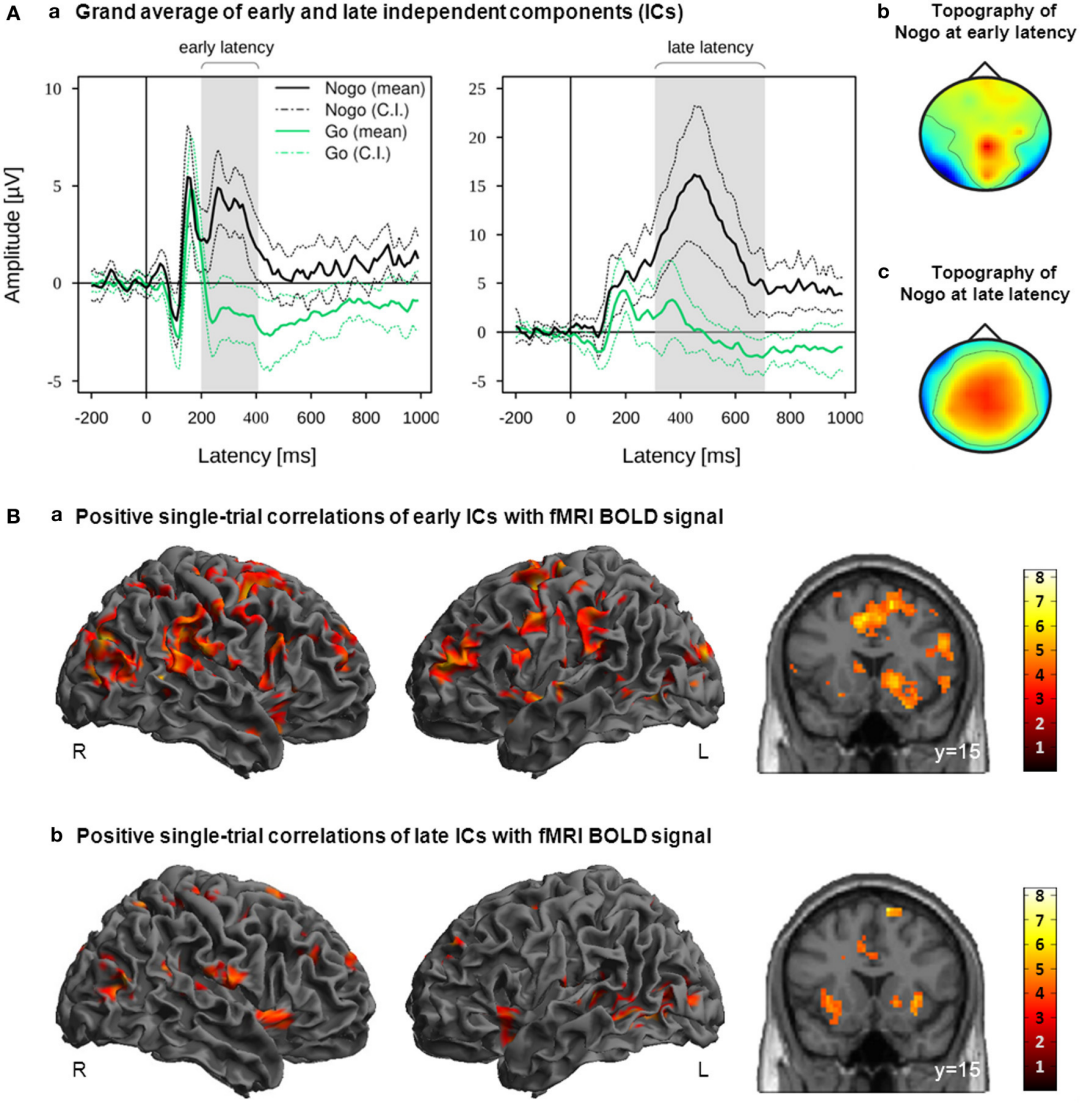
**(A)** IC activation grand averages (a) and grand mean topographies (b,c) of all participants ICs which were classified as reliably Nogo-related within the early latency range or late latency range. Mean and 95% confidence interval (CI) of Nogo (black lines) and Go (green lines) are displayed in solid and dotted lines, respectively; gray bars indicate the early and late latency ranges on the group level. **(B)** Activation maps displaying the main effects of positive correlations with EEG-derived early (a; time window starting 200 ms after stimulus onset and ending with the RT) and late (b; time window starting 100 ms prior to RT and ending 300 ms after RT) regressors. These regressors were orthogonalized to onset regressors. Images are displayed in neurological order (L, Left; R, Right), with *p* < 0.005 (unc.) and *k* = 20. RT, individual median Go response time.

***IC selection.*** To construct regressors representing different phases of the inhibition processes, we combined ICs and latency ranges falling into different time windows (Figure 2). These time windows were defined for each participant in relation to stimulus onset and the individual’s median correct Go RT. To capture neural correlates of an early stage of response inhibition without including correlates of visual processing and object recognition (Johnson and Olshausen, 2003), the “early” time window starts 200 ms after stimulus onset and ends with the individual median RT (Figure 2, yellow part). To capture neural correlates of a later stage of response inhibition we defined a second time window located around the participant’s RT. Considering the trial-to-trial fluctuations of single trial RTs around median RT the “late” time window starts 100 ms before RT and ends 300 ms after RT (Figure 2, red part). We allowed overlapping latency windows because both start and end of each acceptable latency range as determined previously (Nogo-Go |*Z*| > 0.275) were strictly required to fall within the given ranges (Figure 3A). This means that any acceptable activity not only has to be significant within the given time range but must also start (i.e., rise above *Z*-threshold) after the beginning and end (i.e., fall under *Z*-threshold) before the end of the given range. The overlapping time ranges were chosen to avoid losing too many meaningful activity candidates.

***Feature extraction.*** For every selected IC, mean amplitudes of all single trials were extracted from the latency range in which the respective IC component was reliably larger during Nogo and inverted if necessary to ensure positive polarity with respect to the Nogo-Go difference, using the polarity noted above. To be able to compare the time ranges across a single group of participants, participants failing to display Nogo-specific ICs on the chosen absolute *Z*-score level (|*Z*| > 0.275) for any time window were excluded. For each of the remaining 22 participants (7 males; mean age: 34.41 ± 14.1), amplitude vectors of ICs selected with respect to the same time window (early or late) and run were combined into a single amplitude vector by summation. This resulted in two different EEG-derived regressors for each participant and run: early and late.

#### Visual ICs

To assess whether component selection can be done on other electrophysiological components in the same dataset of the visual Go/Nogo task, we modified the approach so that visual components can be detected.

***IC classification.*** In contrast to the Nogo-related ICs which we expected to have larger amplitudes in Nogo than in Go conditions, we assumed that visual responses are similar in Go and Nogo conditions. Thus, instead of for the Nogo-Go difference, IC average and variance were computed across Nogo and Go epochs. ICs were classified as sufficiently reliably activated if their absolute *Z*-score exceeded a threshold of 0.275. For each participant and run, a list of ICs, latency ranges and polarities was formed.

***IC selection.*** To construct a regressor representing visual processing we combined ICs and latency ranges falling into a time window starting 90 ms after stimulus onset and ending 140 ms after stimulus onset (Figure 5A).

***Feature extraction.*** For every selected IC, mean amplitudes of each single trial were extracted from the latency range in which the respective IC component had crossed the predefined *Z*-threshold. Two participants failed to display visual ICs on the chosen absolute *Z*-score level (|*Z*| > 0.275) and were therefore excluded from any further analyses. For each of the remaining 37 participants (15 males; mean age: 38.27 ± 16.1) and run, amplitude vectors of the selected ICs were combined into a single amplitude vector by summation.

### Classification and selection of N2/P3 ERPs

To compare the automated IC-based approach to a more classical approach based on selecting single-trial amplitude values from predefined ERP components, we extracted the mean N2/P3 amplitude values. To achieve improved comparability, single-trial EEG/fMRI analysis of N2/P3 amplitudes was computed for the same 22 participants as the single-trial EEG/fMRI analysis of Nogo-related ICs.

The BCG/EOG artifact corrected EEG of each participant was re-referenced to the average of TP9 and TP10 and segmented into epochs of 1200 ms starting 200 ms prior to stimulus onset. The 200 ms pre-stimulus interval was used for baseline correction. Epochs belonging to the same event type (i.e., correct response: Go and Nogo; incorrect response: omission of Go trials and commission errors in Nogo trials) were averaged, resulting in four different event-related averages.

**Figure 4:**
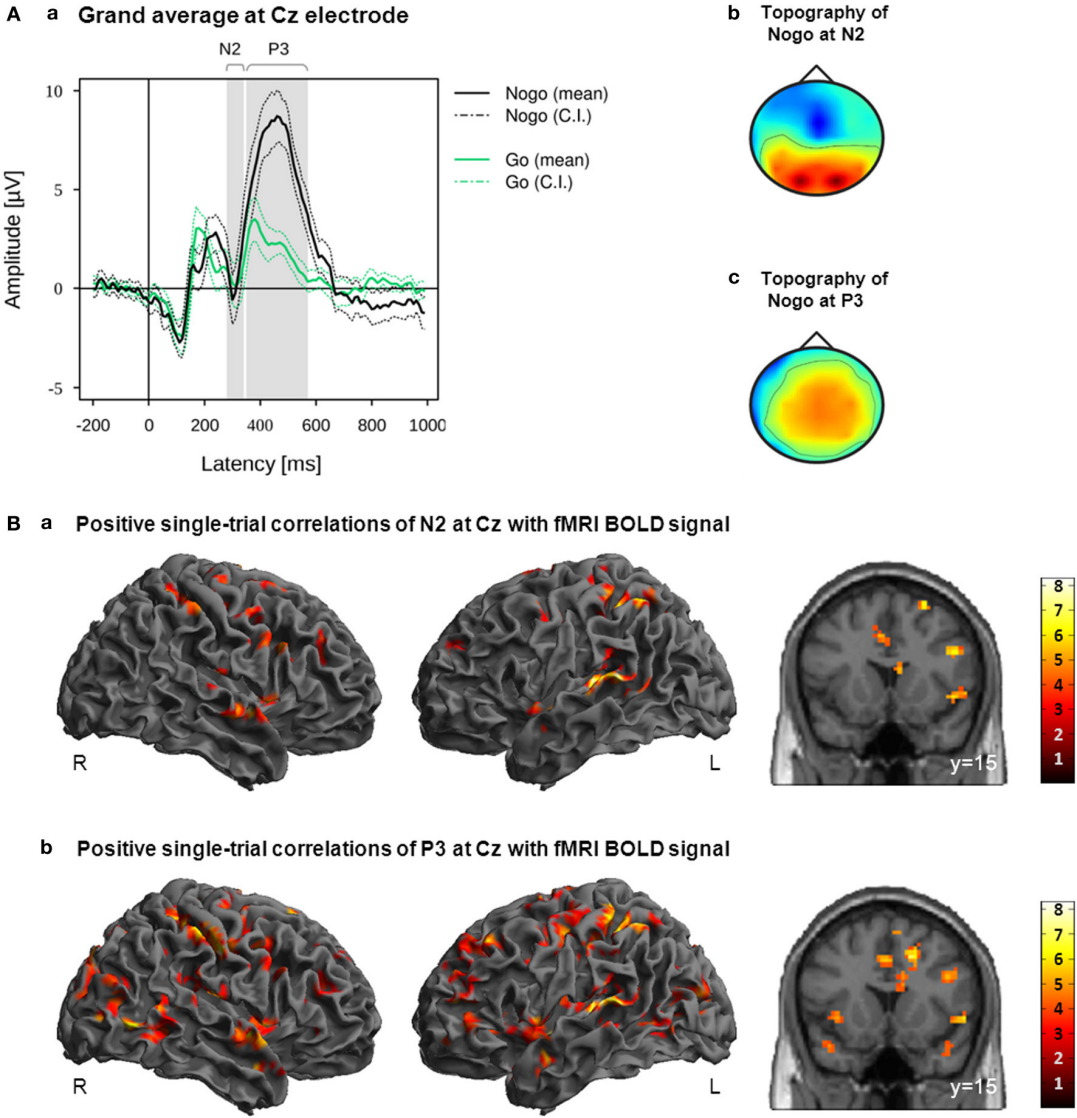
**(A)** Grand average (a) and grand mean topographies at N2 latency range (b) and P3 latency range (c). Mean and 95% confidence interval (CI) of Nogo (black lines) and Go (green lines) are displayed in solid and dotted lines; gray bars indicate the early and late latency ranges on the group level (a). **(B)** Activation maps displaying the main effects of positive correlations with EEG-derived N2 (a; time window 280–340 ms after stimulus onset) and P3 (b; time window 350–570 ms after stimulus onset) regressors. These regressors were orthogonalized to onset regressors. Images are displayed in neurological order (L, Left; R, Right), with *p* < 0.005 (unc.) and *k* = 20.

For each participant, single-trial amplitude values were extracted from Cz where the Nogo-N2/-P3 effects were most pronounced in the grand average. N2 was measured as the mean amplitude in the time window 280–340 ms after stimulus onset, whereas P3 was measured as the mean amplitude between 350 and 570 ms after stimulus. These latency ranges were chosen to cover best the task-related N2 and P3 effects on group level (Figure 4A) Mean amplitudes of each single trial were extracted from the N2 and P3 latency ranges at Cz, resulting in two amplitude vectors for each participant.

### fMRI regressors

To fit the sampling frequencies of EEG-derived regressors (*f* = 1 Hz) and fMRI data acquisition (*f* = 1/2.25 Hz), each joint amplitude vector was interpolated over time by using a cubic smoothing spline function and re-sampled at the time points of fMRI data acquisition. This down-sampled time course was then normalized to inter-quartile range (IQR = 1) and convolved with a canonical hemodynamic response function. In a second step, each EEG-derived regressor was orthogonalized with respect to classical onset regressors (Go, Nogo, Errors).

### First-level single-trial EEG/fMRI analysis

Statistical analysis of fMRI data was performed using SPM8 running under Matlab 7.7.0. For each participant (*N* = 37 in case of visual components and *N* = 22 in all other cases) different GLMs were fitted separately to the fMRI data. The design matrix of each GLM contained four regressors of interest: three onset regressors (Go, Nogo, and Errors) and one EEG regressor derived from either early or late ICs, N2 or P3 ERP, or visual ICs. Task-related EEG-derived regressors were orthogonalized to the onset regressors, whereas the visual regressor was not orthogonalized. The time courses of regressors and functional data were run through a high-pass filter with a 128 s cut-off in order to remove artifacts resulting from low frequency temporal variations. Since ICA was applied to each run independently, we obtained two EEG-derived regressors and thus two corresponding contrast images for each participant and time window. A single beta image per participant was computed for second-level analyses by averaging the two contrast images belonging to the same participant and time window.

### Group analysis

The first level analysis results of task-related regressors (i.e., early/late and N2/P3) were subjected to paired *t*-tests with either early/late or N2/P3 as paired observations. The first level analysis results of EEG regressors derived from visual components were subjected to a one-sample *t*-test. For whole brain analysis the statistic images were assessed for cluster-wise significance by using a cluster-defining height threshold of *p* < 0.05 (family-wise error = FWE correction for multiple comparisons). In the case of region of interest (ROI) analyses, clusters were assessed for peak-wise significance by using a height threshold of *p* < 0.05 (FWE corrected). Following Sebastian et al. (2013b), small volume correction was computed for the following predefined ROIs as derived from the automated anatomical labeling atlas (AAL): lateral inferior frontal cortex (IFC; derived from a combination of pars opercularis and pars triangularis); middle frontal gyrus; pre-supplementary motor area (preSMA; derived from the SMA region with *y* > 0); caudate nucleus; putamen and pallidum. Additionally small volume correction was computed for the subthalamic nucleus (STN), consisting of two boxes of respectively 10 × 10 × 10 mm in size and localized at the MNI coordinates −10, −15, −5 (left STN) and 10, −15, −5 (right STN) (Aron and Poldrack, 2006). For visual components, bilateral inferior occipital cortex, bilateral middle occipital cortex and bilateral superior occipital cortex were additionally included.

## Results

### EEG/fMRI single-trial analysis of nogo-related ICs

Positive correlations of the fMRI BOLD signal with EEG regressors derived from IC related to task processing at an early (200 ms after stimulus onset until individual median RT) and later (100 ms prior to median RT until 300 ms after median RT) stage of response inhibition are listed in Table 1. Due to orthogonalization, these regressors revealed those parts of the trial-to-trial fluctuation that are not captured by the onset regressors. As shown in Figure 3B, EEG-derived regressors correlated positively with fMRI BOLD signal in cortical and subcortical regions associated with response inhibition. Although no significant differences between early and late were found at the level of *p* < 0.05 (FWE corrected), it can be seen that correlations with early and late EEG-derived regressors revealed overlapping but also different areas of activation in regions associated with response inhibition.

**Table 1:** **Positive correlations of fMRI BOLD signal with orthogonalized EEG-derived regressors early (time window starting 200 ms after stimulus onset and ending with the individual median RT) and late (time window starting 100 ms prior to RT and ending 300 ms after RT)**.

**Region**		**Early: positive correlations**						**Late: positive correlations**					
		***x***	***y***	***z***	***k***	***Z***	***p***	***x***	***y***	***z***	***k***	***Z***	***p***
**FRONTAL LOBE**													
IFG (pars opercularis)	R	54	18	27	36	3.84	0.045	–	–	–	–	–	–
IFG (pars orbitalis)	R	51	21	−3	516	4.20	0.013^*^	–	–	–	–	–	–
Middle frontal gyrus	R	24	51	33	821	4.18	0.020^*^	27	36	24	213	4.49	0.005^*^
Middle frontal gyrus	L	−39	45	15	187	4.55	<0.001	−27	42	36	225	4.01	0.038^*^
Superior frontal gyrus	R	24	54	33	92	4.42	<0.001	–	–	–	–	–	–
Superior frontal gyrus	L	−21	−3	57	636	5.37	<0.001	–	–	–	–	–	–
pre-SMA	C	−9	3	48	449	4.46	0.002^*^	12	15	66	132	4.63	0.001^*^
Precentral gyrus	L	−36	3	39	101	4.28	<0.001	–	–	–	–	–	–
**TEMPORAL LOBE**													
Superior temporal gyrus	L	−63	−27	42	86	4.01	0.001	−51	−33	9	54	4.60	0.008
Temporal pole/insula lobe	R	–	–	–	–	–	–	36	12	0	59	4.07	0.005
Middle temporal gyrus	R	51	−72	18	44	4.06	0.020	–	–	–	–	–	–
Supramarginal gyrus	R	66	−39	24	317	5.15	<0.001	66	−21	18	48	4.29	0.014
Insula lobe	L	−39	−6	−6	57	4.49	0.006	–	–	–	–	–	–
Insula lobe/amygdala	R	42	−3	−27	58	4.24	0.005	–	–	–	–	–	–
Insula lobe/amygdala	L	–	–	–	–	–	–	−27	6	−15	56	4.68	0.007
Hippocampus	L	−24	−18	−9	57	4.93	0.006	−27	−15	−12	53	5.24	0.009
**PARIETAL LOBE**													
Precuneus	R	9	−42	54	128	4.68	<0.001	15	−54	60	65	4.70	0.003
**OCCIPITAL LOBE**													
Middle occipital gyrus	R	33	−78	27	146	4.93	<0.001	–	–	–	–	–	–
Middle occipital gyrus	L	–	–	–	–	–	–	−39	−63	0	57	4.79	0.006
Superior occipital gyrus	L	−18	−78	27	217	4.76	<0.001	−24	−66	21	88	4.68	<0.001
Lingual gyrus	L	–	–	–	–	–	–	−3	−72	0	36	4.57	0.045
**SUBCORTICAL AREAS**													
Putamen	R	18	15	−3	45	3.87	0.018	–	–	–	–	–	–
Putamen	L	–	–	–	–	–	–	−27	6	−9	86	3.84	0.017^*^
Caudate nucleus	R	15	15	−3	154	3.85	0.015^*^	–	–	–	–	–	–
Caudate nucleus	L	−9	12	9	103	3.53	0.045^*^	–	–	–	–	–	–
Pallidum	R	15	9	−3	^*^	3.74	0.004^*^	–	–	–	–	–	–
Pallidum	L	−21	0	6	20	3.21	0.023^*^	–	–	–	–	–	–

The region in which the cluster’s local maximum is located in hemispheres Right (R), Left (L), or Central (C); the peak location in MNI coordinates (x, y, z); cluster extend in number of voxels (k); maximum Z-score; and FWE-corrected p-values (cluster level corrected,

* small volume corrected) are reported for each significantly activated cluster separately. IFG, inferior frontal cortex. SMA, supplementary motor area.

The early regressor but not the late regressors correlated positively with fMRI BOLD signal in bilateral frontal regions such as right posterior IFG (pars opercularis), right Insula/IFG (pars orbitalis), bilateral superior frontal gyrus and left precentral gyrus (adjacent to inferior frontal junction) as well as bilateral insula lobe. Subcortically positive correlations between fMRI BOLD signal and early regressor were found in right putamen, bilateral caudate nucleus and bilateral pallidum, whereas the late regressor correlated positively with left putamen. Positive correlations with both regressors but with reduced cluster size in correlations with late regressor were found in pre-SMA, bilateral dorso-lateral prefrontal cortex and right supramarginal gyrus/temporo-parietal junctions. Further positive correlations between fMRI BOLD signal and both EEG-derived regressors were found in left superior temporal gyrus, right precuneus, bilateral occipital regions, left hippocampus and bilateral insula lobe/amygdala.

### EEG/fMRI single-trial analysis of N2/P3 ERPs

Positive and negative correlations of the fMRI BOLD signal with EEG regressors derived from Cz electrode at the latency ranges N2 (280–340 ms post-stimulus) and P3 (350–570 ms post-stimulus) are listed in Tables 2, 3. As these regressors were orthogonalized to onset regressors, correlations of these EEG-derived regressors with fMRI BOLD signal only revealed that part of the trial-to-trial fluctuation that is not captured by the onset regressors. As shown in Figure 4B, the N2/P3 EEG-derived regressors correlated positively with fMRI BOLD signal in cortical and subcortical regions associated with response inhibition. Despite significant differences (P3 > N2) in left postcentral gyrus, left STN/thalamus and a large area stretching from cerebellar vermis/lingual gyrus to cuneus/precuneus (Table 4), it can be seen that correlations with N2 and P3 EEG-derived regressors revealed overlapping but also different areas of activation in regions associated with response inhibition.

**Table 2:** **Positive correlations of fMRI BOLD signal with orthogonalized EEG-derived regressors N2 (280–340 ms after stimulus onset), and P3 (350–570 ms after stimulus onset)**.

**Region**		**N2: positive correlations**						**P3: positive correlations**					
		***x***	***y***	***z***	***k***	***Z***	***p***	***x***	***y***	***z***	***k***	***Z***	***p***
**FRONTAL LOBE**													
IFG (pars opercularis)	R	51	15	27	35	4.24	0.036	–	–	–	–	–	–
IFG (pars orbitalis)	R	51	12	−3	218	4.60	0.002^*^	51	12	−3	53	4.49	0.004^*^
IFG (pars triangularis)	L	–	–	–	–	–	–	−45	45	9	48	4.32	0.009
Middle frontal gyrus	R	–	–	–	–	–	–	27	3	51	215	4.23	0.016^*^
Middle frontal gyrus	L	−30	48	33	117	4.71	0.002^*^	−33	27	45	412	4.74	0.001^*^
pre-SMA	C	6	18	63	101	4.38	0.004^*^	6	18	63	157	4.71	0.001^*^
Precentral gyrus	R	–	–	–	–	–	–	27	−9	48	59	4.65	0.003
Middle cingulate cortex	L	–	–	–	–	–	–	−9	−33	45	41	4.68	0.019
**TEMPORAL LOBE**													
Superior temporal gyrus/insula lobe	R	54	−3	−3	99	5.27	<0.001	–	–	–	–	–	–
Superior temporal gyrus/insula lobe	L	−39	−12	−6	76	5.08	0.001	−39	−12	−6	183	5.93	<0.001
Superior temporal gyrus	L	−42	−27	12	36	5.51	0.032	−42	−27	12	35	5.38	0.036
Superior temporal gyrus	L	−63	−39	12	76	5.20	0.001	−63	−33	15	48	5.78	0.009
Middle/inferior temporal gyrus	R	–	–	–	–	–	–	57	−66	0	44	4.97	0.014
Fusiform gyrus	R	–	–	–	–	–	–	33	−39	−15	74	5.31	0.001
Temporal pole/insula lobe	R	–	–	–	–	–	–	60	3	−9	148	5.41	<0.001
**PARIETAL LOBE**													
Supramarginal gyrus	R	–	–	–	–	–	–	54	−21	18	42	4.49	0.017
Postcentral gyrus	R	–	–	–	–	–	–	51	−30	51	94	5.48	<0.001
Inferior parietal lobule	L	−39	−51	54	80	4.95	<0.001	−42	−51	54	631	5.05	<0.001
Precuneus	R	9	−45	60	63	4.93	0.002	18	−42	57	49	4.45	0.008
**OCCIPITAL LOBE**													
Middle occipital gyrus	R	–	–	–	–	–	–	36	−75	6	46	4.35	0.011
Middle occipital gyrus	L	–	–	–	–	–	–	−27	−69	30	68	4.36	0.001
Lingual gyrus	R	–	–	–	–	–	–	12	−54	−3	33	4.17	0.045
Lingual gyrus	L	−27	−48	−3	43	5.08	0.015	−18	−66	−9	676	5.31	<0.001
Cuneus/precuneus	R	–	–	–	–	–	–	24	−54	30	148	4.97	<0.001
**SUBCORTICAL AREAS**													
Caudate nucleus	L	−18	−15	24	14	3.95	0.010^*^	–	–	–	–	–	–
Putamen	L	–	–	–	–	–	–	−33	−15	−6	14	3.65	0.035^*^
Subthalamic nucleus	L	–	–	–	–	–	–	−12	−18	−6	5	2.92	0.047^*^
Thalamus	R	–	–	–	–	–	–	21	−27	−3	79	4.61	<0.001
Thalamus/hippocampus	L	–	–	–	–	–	–	−21	−24	−6	138	5.50	<0.001

The region in which the cluster’s local maximum is located in hemispheres Right (R), Left (L), or Central (C); peak location in MNI coordinates (x, y, z); cluster extend in number of voxels (k); the maximum Z-score; and FWE-corrected p-values (cluster level corrected,

* small volume corrected) are reported for each significantly activated cluster separately. IFG, inferior frontal cortex. SMA, supplementary motor area.

**Table 3:** **Negative correlations of fMRI BOLD signal with orthogonalized EEG-derived regressor N2 (280–340 ms after stimulus onset)**.

**Region**		**N2: negative correlations**					
		***x***	***y***	***z***	***k***	***Z***	***p***
**FRONTAL LOBE**							
Superior medial gyrus	L	−6	60	3	121	5.00	<0.001
Middle frontal gyrus	L	−27	54	9	48	4.26	0.013^*^
pre-SMA	C	0	18	54	73	3.99	0.020^*^
**TEMPORAL LOBE**							
Fusiform gyrus	R	27	−42	−12	88	5.21	<0.001
Fusiform gyrus	L	−24	−45	−15	47	5.17	0.010
Angular gyrus	L	−42	−75	39	48	4.22	0.009
**OCCIPITAL LOBE**							
Lingual gyrus/cerebellum	R	9	−54	−15	156	4.52	<0.001
**SUBCORTICAL AREAS**							
Caudate nucleus	R	12	−3	18	31	3.60	0.041^*^
Pallidum	R	15	3	3	10	3.68	0.005^*^
Subthalamic nucleus	L	−12	−18	−9	8	3.16	0.021^*^

The region in which the cluster’s local maximum is located in hemispheres right (R), left (L), or central (C); peak location in MNI coordinates (x, y, z); cluster extend in number of voxels (k); the maximum Z-score; and FWE-corrected p-values (cluster level corrected,

* small volume corrected) are reported for each significantly activated cluster separately. SMA, supplementary motor area.

**Table 4:** **Brain regions significantly stronger correlated with P3 single-trial amplitude values (350–570 ms after stimulus onset) than with N2 single-trial amplitude values (280–340 ms after stimulus onset)**.

**Region**		**P3 > N2**					
		***x***	***y***	***z***	***k***	***Z***	***p***
**PARIETAL LOBE**							
Postcentral gyrus	L	−39	−24	54	91	3.45	0.029
**OCCIPITAL LOBE**							
Lingual/calcarine gyrus	C	0	−63	12	414	4.53	<0.001
**SUBCORTICAL AREAS**							
Subthalamic nucleus	L	−12	−18	−6	11	3.49	0.006^*^
Thalamus	L	−18	−24	−6	88	3.61	0.034

The region in which the cluster’s local maximum is located in hemispheres Left (L) or Central (C); peak location in MNI coordinates (x, y, z); cluster extend in number of voxels (k); maximum Z-score; and FWE-corrected p-values (cluster level corrected,

* small volume corrected) are reported for each significantly activated cluster separately.

The N2 regressor but not the P3 regressors correlated positively with fMRI BOLD signal in right posterior IFG (pars opercularis), right superior temporal gyrus and left caudate nucleus. The P3 regressor but not the N2 regressors correlated positively with fMRI BOLD signal in left anterior IFG (pars triangularis), right precentral gyrus, left middle cingulate cortex, right middle and inferior temporal regions, bilateral occipital areas and subcortical regions such as left putamen, left STN and bilateral thalamus/hippocampus. Positive correlations with both regressors but with reduced cluster size in correlations with P3 regressor were found in right Insula/IFG (pars orbitalis) and right precuneus/inferior parietal lobule. Reduced cluster size in correlations with N2 regressor compared to P3 regressor was found in left dorso-lateral prefrontal cortex, right pre-SMA and left superior temporal gyrus. The N2 regressor but not the P3 regressors correlated negatively with fMRI BOLD signal in a cluster located at the superior medial frontal gyrus and a large area stretching from central lingual gyrus and cerebellum to precuneus and calcarine gyrus, but also in smaller cortical and subcortical clusters located in pre-SMA, left middle frontal gyrus, bilateral fusiform gyri, left STN, and right Pallidum and caudate nucleus (Table 3).

Supplementary Table 1 contains a side-by-side comparison of positive fMRI BOLD correlations obtained using the new method (early/late regressors, section EEG/fMRI Single-trial Analysis of Nogo-related ICs) and the classical method (current section).

### EEG/fMRI single-trial analysis of visual ICs

**Figure 5:**
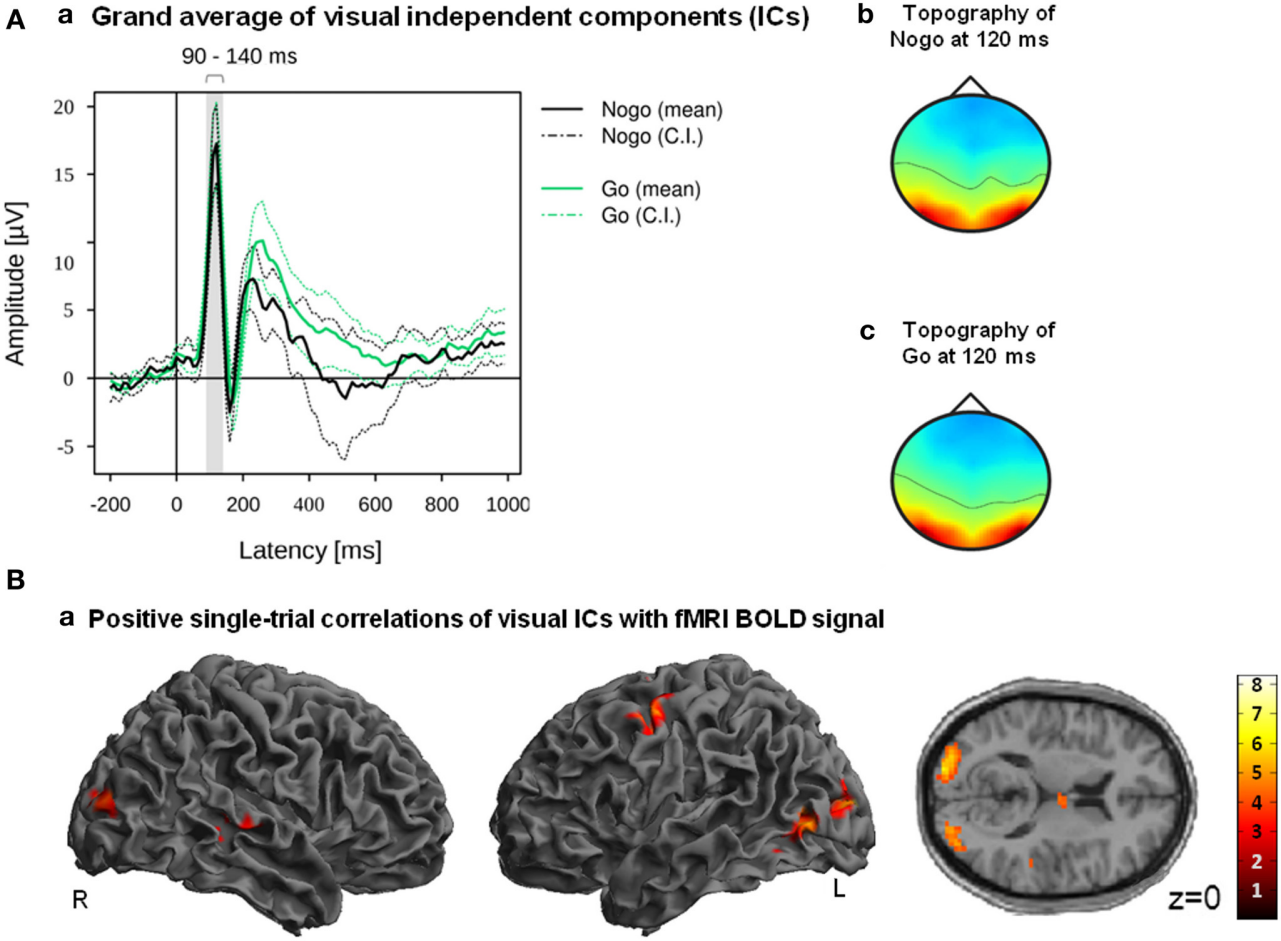
**(A)** Grand averages (a) and grand mean IC topography (b) of all participants ICs which were classified as reliable related to visual processing within the latency range of 90–140 ms. Mean and 95% confidence interval (CI) of Nogo (black lines) and Go (green lines) are displayed in solid and dotted lines; the gray bar indicates the latency range of 90–140 ms (a). **(B)** Positive correlations of fMRI BOLD signal with EEG regressor derived from visual components (90–140 ms after stimulus onset). Images are displayed in neurological order (L, Left; R, Right), with *p* < 0.005 (unc.) and *k* = 20.

Positive correlations of the fMRI BOLD signal with EEG regressors derived from IC related to visual processing (ICs with |*Z*| > 0.275 within the latency ranges of 90–140 ms post-stimulus) are listed in Table 5. Correlations of fMRI BOLD signal with EEG regressor derived from single-trial amplitudes of visual components yielded activations primarily in visual areas but also in the left premotor cortex (Figure 5B). However, significant positive correlations at the level of *p* < 0.05 (FWE corrected) were found exclusively in visual areas (bilateral middle and superior occipital gyri) but not in premotor areas.

**Table 5:** **Positive correlations of fMRI BOLD signal EEG-regressor derived from visual response (time window starting 90 ms after stimulus onset and ending 140 ms after stimulus onset)**.

**Region**		**Visual components**					
		***x***	***y***	***z***	***k***	***Z***	***p***
**OCCIPITAL LOBE**							
Superior occipital gyrus	R	24	−93	12	42	3.65	0.037^*^
Superior occipital gyrus	L	−15	−93	12	70	4.22	0.003^*^
Middle occipital gyrus	R	33	−87	15	114	3.88	0.021^*^
Middle occipital gyrus	L	−21	−93	9	55	5.25	0.019
Middle occipital gyrus	L	−42	−69	3	59	4.69	0.014

The region in which the cluster’s local maximum is located in hemispheres Right (R) or Left (L); peak location in MNI coordinates (x, y, z); cluster extend in number of voxels (k); maximum Z-score; and FWE-corrected p-values (cluster level corrected,

* small volume corrected) are reported for each significantly activated cluster separately.

## Discussion

The current study aimed at a data-driven identification of correlates of trial-to-trial variability in inhibition specific neurophysiological activity in simultaneously acquired EEG and fMRI. Using data of 39 healthy participants in a visual Go/Nogo task, single trial EEG/fMRI analysis was performed based on the automated identification of inhibition-related electrophysiological ICs. This identification was done for each participant in a completely data driven way using an extended ICA (Bell and Sejnowski, 1995; Lee et al., 1999; Makeig et al., 1999, 2002). Specifically Nogo-related ICs (i.e., Nogo minus Go) were identified by *Z*-scores of stimulus-locked averages above a predefined threshold within one of two time windows.

### Single-trial EEG/fMRI analysis of nogo-related ICs and N2/P3 ERPs

In those participants showing reliable inhibition-related components, we were able to analyze the relationship between trial-to-trial variations in these ICs and fMRI brain activity. Due to the high temporal resolution of the EEG, we could specifically assess inhibition-related EEG activity occurring clearly before the typical RT (early time window) and inhibition-related EEG activity occurring around the typical RT (later time window; Figure 3A). The corresponding EEG-derived regressors were orthogonalized to the classical paradigm-derived onset regressors to reveal only those brain regions in which the BOLD signal is attributed genuinely to trial-to-trial fluctuations of inhibition-related ICs rather than to condition effects. Both, early and late EEG-derived regressors correlated positively with fronto-striatal regions (right IFC, pre-SMA and basal ganglia) associated with response inhibition (Chambers et al., 2009; Aron, 2011). Although there were no significant differences between early and late, in most areas including right Insula/IFC, right posterior IFC, premotor areas and basal ganglia, correlations of fMRI BOLD signal with the early EEG regressor were stronger than with the late EEG regressor. This indicates that the strength of positive correlations is decreasing from early to late stages of response inhibition. However, when interpreting results of single-trial EEG/fMRI analysis it should be considered that although the EEG’s high temporal resolution allows extracting electrophysiological activity clearly related to different stages of neural processing, the fMRI BOLD signal’s temporal resolution remains low. Accordingly, we are able to correlate electrophysiological signals generated by the brain at different stages of neural processing with the fMRI BOLD signal but we are not able to distinguish whether there is a causal relationship between a certain region and the Nogo-related activity or whether the activity of the regions are just statistically more likely to be preceded, accompanied, or followed by Nogo-related activity without a causal relationship.

Single-trial correlation of N2/P3 amplitude values with fMRI BOLD signal was computed for the same 22 participants as the single-trial EEG/fMRI analysis of Nogo-related ICs. The N2-dervied EEG regressor correlated negatively with regions associated with the default mode network (precuneus and superior medial cortex) (Raichle et al., 2001; Buckner et al., 2008) but also with areas in pre-SMA, middle frontal gyrus and basal ganglia. To some degree, these results are consistent with Baumeister et al. (2014), who found negative correlations of increased N2 amplitudes in right middle frontal gyrus, bilateral middle temporal and fusiform gyri but also in regions associated with the default mode network (right precuneus, bilateral superior temporal gyrus and right medial frontal gyrus). As discussed by Baumeister et al. (2014), this might indicate an association between increasing N2 amplitudes and deactivation of the default mode network.

Both, N2- and P3-derived EEG regressors correlated positively with fMRI BOLD signal in fronto-striatal regions associated with response inhibition (right IFC, pre-SMA and basal ganglia), but also in distributed areas located in temporal and parietal lobule. Except for right IFC the degree of positive correlations with N2/P3-derived EEG regressors increased from N2 to P3, whereas in IC-based EEG/fMRI analysis fewer regions were positively correlated with the late EEG regressor relative to the early EEG regressor. The latter may indicate that regions relevant for a successful response inhibition are up-regulated at an early stage of response inhibition but not at the later stage of response inhibition. This seems to be reasonable as it could be expected that regions essential for withholding a prepotent motor response are activated prior to the time point when the Go response would be executed. In contrast to this, N2/P3 single-trial amplitude values seem to correlate with a mixture of network parts associated with response inhibition, attentional processing or response monitoring.

When comparing both approaches (i.e., early/late vs. N2/P3) it can be seen that the activation pattern yielded by the early EEG regressor and the N2 EEG regressor are largely deviating. This might be related to fundamental differences in both approaches. While the early and late EEG regressors were constructed exclusively of participant-specific components which are reliably differentiating between different task conditions at the respective latency range, the N2/P3 ERPs were defined on the group level at the latency ranges and EEG site with the most pronounced Nogo effect. Repeated measure ANOVA with the factors condition (Go and Nogo) and ERP (N2 and P3) revealed a significant condition x ERP interaction [*F*_(1, 21)_ = 61.516, *p* < 0.001] on mean amplitude values. However, *post hoc* test revealed that mean amplitudes of P3 but not of N2 were significantly different between Go and Nogo which is in line with Baumeister et al. (2014), who also reported significant differences for P3 amplitude values but not for N2 amplitude values. The prominent difference between correlations of the fMRI BOLD signal with the early EEG regressor or the N2 EEG regressor might be related to the fact that the early ICs are reliably task-discriminating at the respective latency range and thus more sensitive to the Nogo condition, while the N2 seems to be less specific to the task condition.

In conclusion, the deviating results between ERP-based N2/P3 and IC-based early/late single-trial correlations are probably related to the fundamentally different approaches of selecting the EEG features used for single-trial correlations. Following Baumeister et al. (2014), for each participant the mean single-trial amplitude values of N2 and P3 were extracted from Cz electrode at the latency ranges 280–340 and 350–570 ms after stimulus onset. These time windows were chosen as they reflected best the Nogo-N2 and Nogo-P3 effects for the entire group (Figure 4A). However, these fixed time windows were determined on the group level which is insensitive to inter-individual variability as they were observable for example in the participant’s median RT (ranging from 322.81 to 487.94 ms). As it is known that such phenotypes exist even for simple reaction time paradigms, inter-individual differences constitute valuable information when analyzing more complex cognitive functions (Kanai and Rees, 2011). Thus, inter-individual differences make it necessary to verify intra-individually the presence of certain components prior to including them into group-level analyses. Therefore, we developed an analysis procedure that does not build exclusively on N2/P3 effects, but classifies and selects task- and participant-specific electrophysiological components in a completely data driven manner. For every single participant, the algorithm identifies those participant-specific components which are differentiating best between the different task conditions at the specific latency range (Figure 3A). Thus, in contrast to N2/P3 ERPs which were defined on the group level (Figure 4A), early and late regressors were constructed of functionally characterized ICs.

### EEG/fMRI single-trial analysis of visual ICs

We introduced an algorithm that allows for selecting Nogo-related ICs in an automated procedure; however, the fact that the algorithm could identify Nogo-related ICs only in about half of the participants may question the validity of the algorithm. Thus, to test whether our IC selection method is generalizable and usable beyond Nogo-related IC detection, we modified the algorithm so that ICs associated with the visual responses can be detected. Single-trial amplitudes of ICs related to visual processing (i.e., |*Z*| > 0.275 within the latency ranges of 90–140 ms post-stimulus) correlated positively with fMRI BOLD signal in left inferior occipital gyrus as well as bilateral middle and superior occipital gyri.

The results are consistent with Fuglø et al. (2012) who found positive correlations of visual components with fMRI BOLD responses in primary visual cortex and middle occipital gyrus. However, Fuglø et al. (2012) employed a block design with checkerboard stimulus blocks alternating with blocks without stimuli, while continuously estimating VEP amplitudes. Therefore, the resulting regressor necessarily follows the stimulation design to a larger degree. EEG-fMRI correlations from experiments in which either a constant stimulus is repeated or stimuli matched for physical properties such as size, complexity and luminance are presented must be viewed from a different perspective. Variability observed across such stimuli may either reflect early discriminative activity for different stimuli or spontaneous fluctuations in brain state. A study following a spontaneous fluctuation design (visual oddball with motor responses) comparable to the current one revealed positive correlations of visual components with fMRI BOLD signal not only in regions of the visual dorsal stream but also in medial frontal and precentral gyri (Warbrick et al., 2013). In addition to visual cortex activity our approach also revealed medial frontal precentral activity patterns comparable to Warbrick et al. (2013). Thus, despite of different task settings and the fact that Warbrick et al. (2013) used a selection procedure based on a-priori defined ERP components (P1 and N1), whereas we selected ICs related to visual processing in a purely data-driven approach, the resultant correlations between EEG-derived regressors and fMRI BOLD responses are quite similar. This illustrates that beyond of detecting Nogo-related ICs our algorithm is also able to detect ICs related to visual responses in the same data set.

### Limitations

As a result of IC selection only about half of participants could be included into the single-trial EEG/fMRI data analysis. As such these results of temporal signal evolution in response inhibition may not be generalizable. Moreover, the fact that Nogo-related ICs were not identified in one half of the participants suggests that correlations using these restrictive IC selection criteria can only be determined for part of the initial population of a study. On the other hand, the same algorithm was able to detect ICs related to visual responses in 37 of 39 participants, which argues against a principal failure of the algorithm. Thus, the finding that certain Nogo-related components are not present in every participant may give an indication of substantial inter-individual differences as state or trait related differences in cognitive strategy when performing the task, illustrating the sensitivity and specificity of the algorithm for detection of different event related responses.

## Conclusion

Using EEG-derived regressors based on single-trial amplitude variability of Nogo-related ICs selected with respect to different time windows allows visualizing the evolution of brain processes during motor inhibition. Furthermore, by classifying and selecting ICs intra-individually our approach takes account of known inter-individual differences in neural processing. In line with existing automated approaches (Goldman et al., 2009; Wessel and Ullsperger, 2011) we used an algorithm that allows for selecting task-related ICs in an automated procedure. As is the case in the COMPASS-approach (Wessel and Ullsperger, 2011), our approach uses an automated IC selection procedure but is independent of ERP templates, which was one of the major drawbacks of COMPASS. This can be achieved by using an algorithm that automatically selects inhibition-specific ICs with significantly increased amplitude during Nogo trials relative to Go trials. Additionally, these ICs were automatically classified depending on whether the latency range of reliably Nogo-related activity occurred early or late relative to median correct Go RT. This is partially comparable to the approach of Goldman et al. (2009) but without the drawback of just being able to extract one component per time window. As our method is independent of a priori defined ERPs, we suggest that this approach of using functionally defined components could be used for EEG features other than event-related transient responses. Although not tested yet, one possible application would be to use it in the context of background rhythms. In this case, one could selectively use those components that are characterized by increased spectral power in one condition relative to another condition.

### Conflict of interest statement

The authors declare that the research was conducted in the absence of any commercial or financial relationships that could be construed as a potential conflict of interest.
