# Supplementary material for: Data-driven analysis of simultaneous EEG/fMRI using an ICA approach
Source: Front Neurosci. 2014 Jul 1;8:175. doi: 10.3389/fnins.2014.00175 (PMC4077017; doi:10.3389/fnins.2014.00175)
Supplement: Supplementary file 1 [file DataSheet1.PDF]

Supplementary Table: Side-by-side comparison of positive correlations of fMRI BOLD signal with orthogonalized EEG-derived regressors early (time window starting 200 ms after stimulus onset and ending with the individual median RT) and late (time window starting 100 ms prior to RT and ending 300 ms after RT), as well as with orthogonalized EEG-derived regressors N2 (280-340 ms after stimulus onset), and P3 (350-570 ms after stimulus onset).

		**early**						**late**						**N2**						***P3***					
**Region**		**x**	**y**	**z**	**k**	***Z***	***p***	**x**	**y**	**z**	**k**	***Z***	***p***	**x**	**y**	**z**	**k**	***Z***	***p***	**x**	**y**	**z**	**k**	***Z***	***p***
**Frontal Lobe**																									
IFG (pars Opercularis)	R	54	18	27	36	3.84	.045	-	-	-	-	-	-	51	15	27	35	4.24	.036	-	-	-	-	-	-
IFG (pars Orbitalis)	R	51	21	-3	516	4.20	.013*	-	-	-	-	-	-	51	12	-3	218	4.60	.002*	51	12	-3	53	4.49	.004*
IFG (pars Triangularis)	L	-	-	-	-	-	-	-	-	-	-	-	-	-	-	-	-	-	-	-45	45	9	48	4.32	.009
Middle Frontal Gyrus	R	24	51	33	821	4.18	.020*	27	36	24	213	4.49	.005*	-	-	-	-	-	-	27	3	51	215	4.23	.016*
Middle Frontal Gyrus	L	-39	45	15	187	4.55	<.001	-27	42	36	225	4.01	.038*	-30	48	33	117	4.71	.002*	-33	27	45	412	4.74	.001*
Superior Frontal Gyrus	R	24	54	33	92	4.42	<.001	-	-	-	-	-	-	-	-	-	-	-	-	-	-	-	-	-	-
Superior Frontal Gyrus	L	-21	-3	57	636	5.37	<.001	-	-	-	-	-	-	-	-	-	-	-	-	-	-	-	-	-	-
pre-SMA	C	-9	3	48	449	4.46	.002*	12	15	66	132	4.63	.001*	6	18	63	101	4.38	.004*	6	18	63	157	4.71	.001*
Precentral Gyrus	L	-36	3	39	101	4.28	<.001	-	-	-	-	-	-	-	-	-	-	-	-	27	-9	48	59	4.65	.003
Middle Cingulate Cortex	L	-	-	-	-	-	-	-	-	-	-	-	-	-	-	-	-	-	-	-9	-33	45	41	4.68	.019
**Temporal Lobe**																									
Superior Temporal Gyrus	L	-63	-27	42	86	4.01	.001	-51	-33	9	54	4.60	.008	-63	-39	12	76	5.20	.001	-63	-33	15	48	5.78	.009
Superior Temporal Gyrus	L	-	-	-	-	-	-	-	-	-	-	-	-	-42	-27	12	36	5.51	.032	-42	-27	12	35	5.38	.036
Middle/Inferior Temporal Gyrus	R	-	-	-	-	-	-	-	-	-	-	-	-	-	-	-	-	-	-	57	-66	0	44	4.97	.014
Temporal Pole/Insula Lobe	R	-	-	-	-	-	-	36	12	0	59	4.07	.005	-	-	-	-	-	-	60	3	-9	148	5.41	<.001
Middle Temporal Gyrus	R	51	-72	18	44	4.06	.020	-	-	-	-	-	-	-	-	-	-	-	-	-	-	-	-	-	-
Fusiform Gyrus	R													-	-	-	-	-	-	33	-39	-15	74	5.31	.001
Supramarginal Gyrus	R	66	-39	24	317	5.15	<.001	66	-21	18	48	4.29	.014	-	-	-	-	-	-	54	-21	18	42	4.49	.017
Insula Lobe	L	-39	-6	-6	57	4.49	.006	-	-	-	-	-	-	-	-	-	-	-	-	-	-	-	-	-	-
Superior Temporal Gyrus/Insula Lobe	R	-	-	-	-	-	-	-	-	-	-	-	-	54	-3	-3	99	5.27	<.001	-	-	-	-	-	-
Superior Temporal Gyrus/Insula Lobe	L	-	-	-	-	-	-	-	-	-	-	-	-	-39	-12	-6	76	5.08	.001	-39	-12	-6	183	5.93	<.001
Insula Lobe/Amygdala	R	42	-3	-27	58	4.24	.005	-	-	-	-	-	-	-	-	-	-	-	-	-	-	-	-	-	-

Insula Lobe/Amygdala	L	-	-	-	-	-	-	-27	6	-15	56	4.68	.007	-	-	-	-	-	-	-	-	-	-	-	-
Hippocampus	L	-24	-18	-9	57	4.93	.006	-27	-15	-12	53	5.24	.009	-	-	-	-	-	-	-	-	-	-	-	-
**Parietal Lobe**																									
Postcentral Gyrus	R	-	-	-	-	-	-	-	-	-	-	-	-	-	-	-	-	-	-	51	-30	51	94	5.48	<.001
Inferior Parietal Lobule	L	-	-	-	-	-	-	-	-	-	-	-	-	-39	-51	54	80	4.95	<.001	-42	-51	54	631	5.05	<.001
Precuneus	R	9	-42	54	128	4.68	<.001	15	-54	60	65	4.70	.003	9	-45	60	63	4.93	.002	18	-42	57	49	4.45	.008
**Occipital Lobe**																									
Middle Occipital Gyrus	R	33	-78	27	146	4.93	<.001	-	-	-	-	-	-	-	-	-	-	-	-	36	-75	6	46	4.35	.011
Middle Occipital Gyrus	L	-	-	-	-	-	-	-39	-63	0	57	4.79	.006	-	-	-	-	-	-	-27	-69	30	68	4.36	.001
Superior Occipital Gyrus	L	-18	-78	27	217	4.76	<.001	-24	-66	21	88	4.68	<.001	-	-	-	-	-	-	-	-	-	-	-	-
Lingual Gyrus	R	-	-	-	-	-	-	-	-	-	-	-	-	-	-	-	-	-	-	12	-54	-3	33	4.17	.045
Lingual Gyrus	L	-	-	-	-	-	-	-3	-72	0	36	4.57	.045	-27	-48	-3	43	5.08	.015	-18	-66	-9	676	5.31	<.001
Cuneus/Precuneus	R	-	-	-	-	-	-	-	-	-	-	-	-	-	-	-	-	-	-	24	-54	30	148	4.97	<.001
**Subcortical Areas**																									
Putamen	R	18	15	-3	45	3.87	.018	-	-	-	-	-	-	-	-	-	-	-	-	-	-	-	-	-	-
Putamen	L	-	-	-	-	-	-	-27	6	-9	86	3.84	.017*	-	-	-	-	-	-	-33	-15	-6	14	3.65	.035*
Caudate Nucleus	R	15	15	-3	154	3.85	.015*	-	-	-	-	-	-	-	-	-	-	-	-	-	-	-	-	-	-
Caudate Nucleus	L	-9	12	9	103	3.53	.045*	-	-	-	-	-	-	-18	-15	24	14	3.95	.010*	-	-	-	-	-	-
Pallidum	R	15	9	-3	*	3.74	.004*	-	-	-	-	-	-	-	-	-	-	-	-	-	-	-	-	-	-
Pallidum	L	-21	0	6	20	3.21	.023*	-	-	-	-	-	-	-	-	-	-	-	-	-	-	-	-	-	-
Subthalamic Nucleus	L	-	-	-	-	-	-	-	-	-	-	-	-	-	-	-	-	-	-	-12	-18	-6	5	2.92	.047*
Thalamus	R	-	-	-	-	-	-	-	-	-	-	-	-	-	-	-	-	-	-	21	-27	-3	79	4.61	<.001
Thalamus/Hippocampus	L	-	-	-	-	-	-	-	-	-	-	-	-	-	-	-	-	-	-	-21	-24	-6	138	5.50	<.001

The region in which the cluster's local maximum is located in hemispheres right (R), left (L), or central (C); the peak location in MNI coordinates (x, y, z); cluster extend in number of voxels (k); maximum *Z*-score; and FWE-corrected *p*-values (cluster level corrected, * small volume corrected) are reported for each significantly activated cluster separately. IFG: inferior frontal cortex. SMA: supplementary motor area.
